# Changes in central hemodynamics and heart rate variability across active and placebo phases in second‐generation oral contraceptive pill users

**DOI:** 10.14814/phy2.71175

**Published:** 2026-11-09

**Authors:** Jenna C. Stone, Joëlle Chackal, Matthew I. Badour, Maureen J. MacDonald

**Affiliations:** ^1^ Vascular Dynamics Lab, Department of Kinesiology McMaster University Hamilton Ontario Canada

**Keywords:** autonomic function, heart rate variability, oral contraceptive pill, women's health

## Abstract

Heart rate variability (HRV) is a popular, non‐invasive measure of cardiovagal tone. Some research has found that HRV increases from the follicular to the luteal phase of the menstrual cycle, however limited studies investigate the oral contraceptive pill (OCP) cycle. Therefore, the purpose of this study was to determine the influence of second‐generation OCP (OCP2) phase on HRV in the laboratory using electrocardiogram, and in a free‐living situation using the OURA® smart ring. 19 females using an OCP2 underwent measurements of HRV in the morning during the placebo and active phase of their OCP2 cycle and wore an OURA® smart ring. All HRV parameters, including those in the time and frequency domain, were lower in the active phase (all *p* < 0.05) however, these differences did not persist after covarying for resting HR (all *p* > 0.05), except for ECG‐derived root mean square of successive differences and the standard deviation of successive differences. Diastolic and mean arterial blood pressure were lower in the active phase compared to the placebo phase (*p* < 0.05). Therefore, we conclude that HRV is lower in the active phase compared to the placebo phase of the OCP2 cycle, reflecting the integrative regulation of cardiovascular homeostasis.

## 1 INTRODUCTION

Heart rate variability (HRV) is known to be an estimation of cardiovagal tone, which is an index of parasympathetic nervous system activity (Task Force of the European Society of Cardiology the North American Society of Pacing Electrophysiology, 1996). HRV represents the timing variation between successive heart cycles, and therefore reflects largely parasympathetic nervous system contributions to heart rate (HR) (Sztajzel, 2004).

HRV can be represented using a variety of different indices, which are classified into either the “frequency” or “time” domain. Measuring HRV in the frequency domain involves quantifying the contribution from low‐frequency (LF) (0.04–0.15 Hz) and high‐frequency (HF) (0.15–0.40 Hz) components (Montano et al., 2009), and the resultant LF/HF ratio can be used as an estimation of cardiac vagal modulation (Heathers, 2014). Measuring HRV in the time domain refers to measurements related to the sequence of R‐R intervals [i.e., the time interval between consecutive R waves of the electrocardiogram (ECG)] and numerous variables can be extracted such as the root mean square of successive differences (rMSSD), standard deviation of R‐R intervals (SDNN), and the percentage of successive normal R‐R intervals that differ by more than 50 milliseconds (ms) (pNN50) (Sztajzel, 2004). These indices are typically derived from measures conducted using an ECG, which remains the gold standard (Task Force of the European Society of Cardiology the North American Society of Pacing Electrophysiology, 1996). More recently, wearable devices using photoplethysmography (PPG), such as the OURA® smart ring, have enabled reliable estimation of nocturnal HRV, particularly RMSSD, during sleep (Cao et al., 2022). The combination of ECG‐based recordings and wearable technology allows for both controlled laboratory assessments and continuous real‐world monitoring of HRV.

Fluctuations in HRV throughout the natural menstrual cycle have been consistently reported, with higher rMSSD and lower LF/HF ratio in the early follicular phase compared to the luteal phase (Blake et al., 2023; Brar et al., 2015; Ramesh et al., 2022; Schmalenberger et al., 2020). It has been hypothesized that estradiol might enhance parasympathetic activity while progesterone reduces it (Mei et al., 2026). However, not all females experience a natural menstrual cycle. 151 million women worldwide are use oral contraceptive pills (OCPs) (Johansson et al., 2023). There are four generations of OCPs, all differing in hormone type and concentration, with second‐generation OCPs (OCP2) the most commonly used in Canada in 2025 (36.9% of OCP users) (Statistics Canada, 2025). OCP2s contain a combination of levonorgestrel, as synthetic progesterone, and ethinyl estradiol, as synthetic estrogen (Sitruk‐Ware, 2004). Unlike the natural menstrual cycle where hormone levels are continuously fluctuating, OCP use suppresses endogenous hormone concentration, while adding synthetic progesterone (i.e., progestin) and estrogen (i.e., ethinyl estradiol) (Shearman, 1984). Despite OCPs being prescribed in Canada since 1969 (Liao & Dollin, 2012), the influence of OCPs on HRV is unknown.

Some evidence suggests potential differences in HRV between OCP users and naturally cycling females (Pereira et al., 2023). However, most studies report no differences in LF/HF, rMSSD, pNN50, or SDNN, between active (i.e., Days 1–21 of the OCP cycle), and placebo (i.e., Days 22–28 of the OCP cycle) phases. Importantly, these studies do not control, or report for generation of OCP, which may mask progestin‐specific actions (Teixeira et al., 2015; Wilczak et al., 2013). One study observed parasympathetic dominance in the placebo phase of the OCP cycle, using both time, and frequency domains of HRV, however the sample size was small, and again, they did not report OCP generation (Blake et al., 2023).

Considering OCP generation in study design is important because progestins differ in androgenicity. For example, levonorgestrel is a highly androgenic progestin, while other progestins such as cyproterone acetate are antiandrogenic (Stone & MacDonald, 2023). Androgens, like testosterone, have been shown to influence sympathetic nervous system neurotransmitters (Dart, 2002). Therefore, it is plausible that different OCPs may exert divergent effects on cardiovagal tone depending on their androgenic profile. However, to date, this has not been examined, and existing studies do not stratify by OCP type or even report OCP generation.

The purpose of this study was to investigate differences in HRV measured using both the OURA® smart ring and ECG across the active and placebo phase of the OCP2 cycle. We hypothesized that both time domain and frequency domain metrics of HRV will be higher in the placebo phase compared to the active phase in OCP2 users, reflecting the androgenic influence of levonorgestrel during the active phase.

## 2 MATERIALS AND METHODS

### 2.1 Participant recruitment and ethics

Nineteen pre‐menopausal females using an OCP2 for ≥3 months between the ages of 18–35 were recruited from McMaster University and the surrounding Hamilton area via advertisement. OURA® smart ring data was only available for sixteen participants. Volunteers were excluded if they were smokers, had a history of cardiovascular, metabolic, or musculoskeletal diseases, or previously underwent reproductive surgery. All participants provided written informed consent for experimental procedures. This study was approved by the Hamilton Integrated Research Ethics Board (Project ID 16823) and conducted in accordance with the Declaration of Helsinki.

Sample size calculations were determined using R Studio (version 4.40). It was determined that 16 participants were required to achieve 80% power with an alpha criterion set to 0.05 and an effect size of 0.75, determined using HRV specific effect size guidelines (Quintana, 2017).

### 2.2 Experimental study protocol

All experimental visits took place in the Vascular Dynamics Laboratory at McMaster University. Participants took part in two experimental visits: (1) during the placebo week of their OCP2 cycle (Days 1–7), and (2) during the third week of the active phase of their OCP2 cycle (Days 21–28). Visits were counterbalanced in order. Participants were fitted with an OURA® smart ring to wear daily on the second finger of their non‐dominant hand (or fourth finger when the second was not possible) for the duration of these weeks.

Participants were instructed to arrive following a 6‐h fast, after abstaining from alcohol and caffeine for 8‐h and avoiding vigorous physical activity for 24‐h prior to the data collection visits. Otherwise, participants were instructed to maintain normal dietary and physical activity patterns throughout the experimental weeks, while wearing the OURA® smart ring. All data collection visits occurred in the morning between 07:00 and 12:00. Upon arrival to the laboratory participants lay supine for a 10‐min rest period and were instrumented with a single‐lead ECG, and finger blood pressure device calibrated to heart level for continuous blood pressure monitoring on the left hand (Finometer MIDI, Finapres Medical Systems, Amsterdam, The Netherlands). Then, participants were left to rest in silence for a 5‐min period, which was subsequently used for determination of ECG derived HRV parameters. Following this period, resting hemodynamic parameters including systolic blood pressure, diastolic blood pressure, mean arterial pressure, and HR were measured in triplicate using a semi‐automated oscillometric blood pressure machine (Dinamap Carescape V100; GE Healthcare, Mississauga, ON, Canada), with the last two measurements averaged. HR derived from the Dinamap will be referred to as discrete HR. Then, 8 mL of serum was drawn from an antecubital vein, and subsequently centrifuged ≥45 min later at 1000 *g* at 4°C for 10 min and stored at −70°C. Both experimental visits were identical and occurred within 2 h of the same time of day.

### 2.3 Blood hormone analysis

Serum estradiol and progesterone concentrations were measured by the Hamilton Health Sciences Core laboratories using the VITROS Immunodiagnostic Products Estradiol Reagent Pack (cat. no. 855 2630; Ortho Vitros Microwell) and the VITROS Immunodiagnostic Products Progesterone 2 Reagent Pack (cat. no. 619 9454), respectively, on a VITROS [Eci/ECiQ/3600/5600/XT 7600] Immunodiagnostic System. Levonorgestrel assays were conducted in duplicate using a competitive ELISA kit (ThermoFisher Scientific, Catalog #EIALNGC) following manufacturer guidelines. Two participants had levonorgestrel levels during the placebo phase that were below the lowest range of detection in the chemiluminescent assay of 6.25 pg/mL and therefore have been assumed as 0 pg/mL.

### 2.4 Nocturnal heart rate variability measured using the OURA® smart ring

Nocturnal HRV was represented by rMSSD, acquired from the OURA® smart ring. OURA® estimates inter‐beat‐intervals (IBI) using PPG (Fs 250 Hz) using infrared light (900 nm) during sleep. The OURA® smart ring categorizes each IBI as “normal” or “abnormal” using median filters. An abnormal beat deviates more than 16 bpm from the 7‐point median IBI in its immediate neighborhood. An IBI is included in the calculation if five consecutive IBI values are labeled as normal. Each five‐minute sample is processed if a minimum of 30% of intervals are accepted by this criterion. Lastly, the OURA® smart ring algorithm determines average HR and rMSSD for each five‐minute segment and the whole night average. This data was downloaded via Bluetooth to the associated OURA® smartphone application and viewed on the OURA® web platform (Kinnunen, 1997; Kinnunen et al., 2020). The last 6 days of each phase were used, with a minimum of 3 days included, when data for all days were unavailable. rMSSD data from the night preceding each laboratory visit was also extracted as a separate variable.

### 2.5 Morning heart rate variability measured using electrocardiography

Morning HRV was measured in each participant on one occasion during each of the active and placebo phases using a single‐lead ECG, recorded at a sampling frequency of 1000 Hz for 5 min with the participant in the supine position. rMSSD, SDNN, pNN50, standard deviation of successive differences (SDSD), and LF/HF ratio (Task Force of the European Society of Cardiology the North American Society of Pacing Electrophysiology, 1996), were calculated using a commercially available algorithm (LabChart8 Pro, ADInstruments, Colorado Springs, CO, USA). Resting HR was also recorded during this time and will be referred to as continuous HR.

### 2.6 Statistical analysis

All statistical analysis was performed in “R Studio” (version 4.4.0). Data were assessed for normality using the Shapiro–Wilk test, and visual inspection of histogram and Q‐Q plots. Normally distributed variables were analyzed using paired *t*‐tests, while non‐normally distributed variables were analyzed using the Wilcoxon signed‐rank test. Effect sizes are calculated using Cohen's d for normally distributed variables and rank‐biserial correlation (*r*_rb_). Variables followed a normal distribution, unless stated otherwise, in the results section. To account for the influence of HR on HRV, continuous HR was included as a covariate in a linear mixed‐effects model that included a fixed effect of phase and participant as a random intercept. α criterion was set to *p* < 0.05, and all *p* values are reported to three significant figures, and effect sizes are reported to two decimal places. Data are reported as mean ± SD.

## 3 RESULTS

### 3.1 Participant characteristics

Participant characteristics are shown in Table 1. All participants were currently using a monophasic OCP2, which contains levonorgestrel and ethinyl estradiol [Alysena/Avaine (100 mcg levonorgestrel and 20 mcg of ethinyl estradiol: *n* = 18; 150 mcg levonorgstrel and 30 mcg of ethinyl estradiol: *n* = 1)]. Sleep variables measured using the OURA® smart ring were also not different between phases (Supplemental Table 1).

**TABLE 1:** Participant characteristics.

Age (years)	Height (cm)	Weight (kg)	BMI (kg/m^2^)
24 ± 5	161.5 ± 6.9	69.8 ± 20.1	26.7 ± 7.7

*Note*: Data are expressed as mean ± SD. *n* = 19.

Abbreviation: BMI, body mass index.

### 3.2 Serum sex hormones

Serum and levonorgestrel concentrations were non‐normally distributed. Paired serum estradiol concentrations differed, with lower concentrations in the active phase, compared to the placebo phase (*p* = 0.00584). There was no difference in serum progesterone concentrations between phases (*p* = 0.938). Serum levonorgestrel concentrations were higher in the active phase compared to the placebo phase (*p* < 0.001) (Table 2).

**TABLE 2:** Estradiol (pmol/L), progesterone (nmol/L), and levonorgestrel (pg/mL) concentrations in the active and placebo phase of the oral contraceptive pill cycle.

	Placebo	Active	*p*	*d*/*r*_rb_
Estradiol (pmol/L)^#^	130 ± 49	96 ± 29	**0.00584**	0.64
Progesterone (ng/L)	4.0 ± 1.3	4.0 ± 1.2	0.938	0.02
Levonorgestrel (pg/mL)^#^	202.12 ± 180.386	1604.45 ± 1213.70	**<0.001**	−0.88

*Note*: Data are expressed as mean ± SD. Sample sizes were *n* = 19 for estradiol and progesterone, *n* = 18 for levonorgestrel during the placebo phase, and *n* = 17 for levonorgestrel during the active phase. Boldface indicates statistically significant values (*p* < 0.05). Non‐normally distributed data are represented with a ^#^.

### 3.3 Resting hemodynamics

There was no difference in systolic blood pressure (SBP) between phases (*p* = 0.221). Diastolic blood pressure (DBP) and mean arterial pressure (MAP) were non‐normally distributed. The paired values differed between the active and placebo phase, with lower DBP and MAP in the active phase compared to the placebo phase (*p* = 0.00304; *p* = 0.0230, respectively). Pulse pressure (PP) was lower in the active phase compared to the placebo phase (*p* = 0.00241). Discrete and continuous HR were higher in the active phase compared to the placebo phase (*p* = 0.0221; *p* = 0.0317, respectively) (Table 3).

**TABLE 3:** Resting, supine, hemodynamics.

	Placebo	Active	*p*	*d*/*r*_rb_
SBP (mmHg)	110 ± 8	109 ± 8	0.221	0.29
DBP (mmHg)^#^	67 ± 7	63 ± 5	**0.00304**	0.69
MAP (mmHg)^#^	83 ± 7	80 ± 6	**0.0230**	0.53
PP (mmHg)	44 ± 6	47 ± 5	**0.00241**	−0.81
Discrete HR (bpm)	63 ± 12	67 ± 9	**0.0221**	−0.57
Continuous HR (bpm)	66 ± 11	71 ± 10	**0.0317**	−0.53

*Note*: Data are expressed as mean ± SD. *n* = 19. Non‐normally distributed data are represented with a ^#^. Boldface indicates statistically significant values (*p* < 0.05).

Abbreviation: bpm, beats per minute; DBP, diastolic blood pressure; HR, heart rate; MAP, mean arterial pressure; mmHg, millimeters of mercury; PP, pulse pressure; SBP, systolic blood pressure.

### 3.4 Heart rate variability

Morning ECG‐derived rMSSD was not normally distributed, and lower in the active phase than the placebo phase (placebo: 87.14 ± 51.43; active: 51.55 ± 25.77 ms; *p* = 0.00334; *r*_rb_ = 0.65; Figure 1a). After covarying for continuous HR, given that continuous HR was negatively associated with ECG‐derived rMSSD (β =− 2.58, *p* < 0.001), ECG‐derived rMSSD remained different between phases (*p*_adj_ = 0.0287; Figure 1b).

**FIGURE 1:**
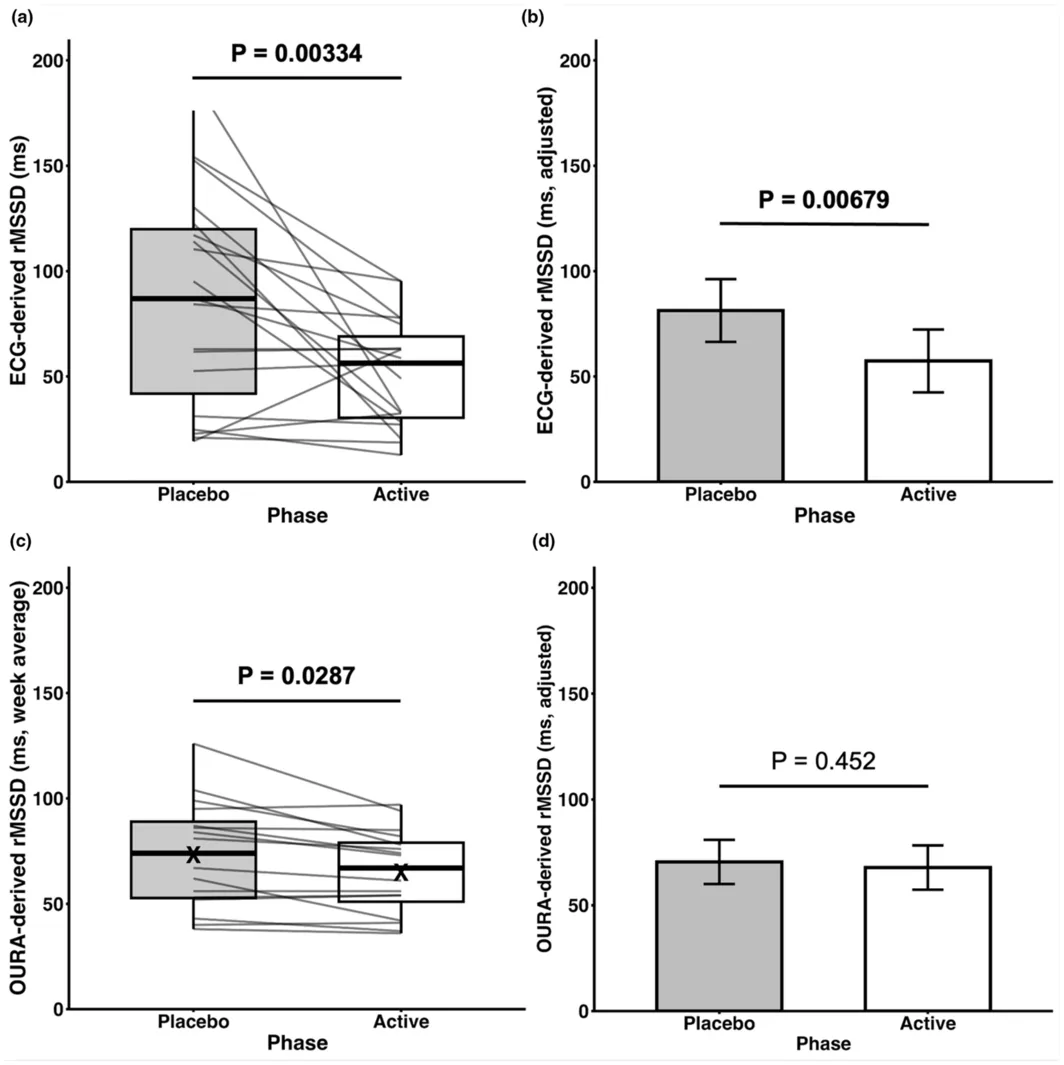
Root mean square of successive differences (rMSSD) is lower in the active phase of the second‐generation oral contraceptive pill (OCP2) cycle compared to the placebo phase and correlated with resting continuous HR. (a) Electrocardiogram (ECG)‐derived rMSSD (ms) measured in the morning. (b) ECG‐derived rMSSD (ms) estimated marginal means after adding continuous resting heart rate (HR) as a fixed effect to a linear mixed effects model. (c) OURA®‐derived rMSSD measured during sleep, averaged across each OCP cycle. (d) OURA®‐derived rMSSD measured during sleep, averaged across the OCP cycle estimated marginal means after adding continuous resting heart rate as a fixed effect to a linear mixed effects model. Box and whisker plots show the median (horizontal line), interquartile range (box), and minimum and maximum values (whiskers). The X indicates the mean, only shown in normally distributed variables. Individual participant values are connected by line across phase. Bars represent estimated marginal means adjusted for continuous heart rate, with error bars representing 95% confidence intervals. Boldface indicates statistically significant values (*p* < 0.05).

Nocturnal OURA®‐derived rMSSD averaged across the week was lower in the active phase compared to the placebo phase (placebo: 73 ± 26; active: 65 ± 20; *p* = 0.00679; *d* = 0.78; Figure 1c). After covarying for continuous HR (β =− 0.872, *p* = 0.0116), nocturnal OURA®‐derived rMSSD averaged across the week was no longer different between phases (*p*_adj_ = 0.452; Figure 1d).

Secondary measures of HRV including SDNN, SDSD, pNN50, and HF, measured using ECG, were not normally distributed, and lower in the active phase compared to the placebo phase (*p* = 0.00392; *p* = 0.0287; *p* = 0.00618; *p* = 0.00405, respectively), while LF/HF was higher in the active phase compared to the placebo phase (*p* = 0.00240). All secondary HRV outcomes showed a significant relationship with continuous HR (all *p* < 0.05). After adding continuous HR as a fixed effect to the model, only the difference between phases for SDSD remained statistically significant (*p*_adj_ = 0.0287) (Table 4).

**TABLE 4:** All HRV parameters extracted in the morning from electrocardiogram using LabChart.

	Placebo	Active	*p*	d/*r*_rb_	*p* (adj)
SDNN (ms)^#^	81.59 ± 38.42	58.46 ± 21.18	**0.00392**	0.64	0.0819
pNN50 (%)^#^	42.25 ± 27.16	27.75 ± 21.73	**0.00618**	0.61	0.251
LF/HF^#^	0.70 ± 0.57	1.08 ± 0.77	**0.00240**	−0.67	0.0578
HF (ms^2^)	3170.6 ± 2966.7	1414.8 ± 1258.4	**0.00405**	0.67	0.0656
SDSD (ms)^#^	87.29 ± 51.52	51.62 ± 25.81	**0.00334**	065	**0.0287**

*Note*: Data are expressed as mean ± SD. *n* = 19. Non‐normally distributed data are represented with a ^#^. *p* (adj) refers to the adjusted *p* value after covarying for continuous heart rate. Boldface indicates statistically significant values (*p* < 0.05).

Abbreviations: LF/HF, power in the low‐frequency band to the high‐frequency band; pNN50, percentage of normal R‐R intervals differing by more than 50 ms; SDNN, standard deviation of all normal R‐R intervals; SDSD, standard deviation of the differences between adjacent R‐R intervals.

## 4 DISCUSSION

We observed lower HRV in the active versus placebo phase of the OCP2 cycle when measured both using the OURA® smart ring at night or by ECG in the morning. However, this relationship was no longer significant after covarying for continuous resting HR, which was higher in the active phase, in all HRV measures other than ECG‐derived rMSSD and SDSD. DBP and MAP were also lower in the active phase compared to the placebo phase of the OCP2 cycle. Therefore, we conclude that the differences in HRV between OCP2 phases could be partially mediated by changes in central hemodynamics.

Our study found that ECG‐derived rMSSD, SDSD, SDNN, and pNN50 were lower, and LF/HF was higher in the active versus placebo phase, prior to covarying for continuous HR. Noctural OURA®‐derived rMSSD was also lower in the active versus placebo phase. The previous literature is mixed with respect to the influence of OCP phase on HRV, likely due to the lack of specific reporting of OCP generation and inclusion of multiple generations without stratification (Pereira et al., 2023). A recent systematic review identified that OCP users exhibit lower HRV than natural cycling females (De Jager et al., 2025). Our study did not include a group of naturally cycling females as a comparator, although this should be considered in future studies. However, our findings suggest that differences in HRV between OCP phases could reflect differences in resting central hemodynamics, given that we observed lower DBP and MAP in the active phase. The HRV findings could be explained by baroreflex control, such that HR was elevated to maintain DBP and MAP (Mancia et al., 1986). It is also possible that the active phase of the OCP2 cycle is associated with a degree of vasodilation, which could have resulted in the modest decrease in DBP and MAP observed and thus an increase in HR to maintain cardiovascular homeostasis. Thus, these changes in HRV may not represent meaningful changes in cardiovagal tone, nor imply an adverse response.

Importantly, to our knowledge, research examining changes in HRV in females using OCPs has not used resting HR as a covariate (Ahokas et al., 2023), or reported resting HR (Blake et al., 2023; Teixeira et al., 2015; Wilczak et al., 2013). This may partially explain the previous finding that HRV is lower in the high hormone phase compared to the low hormone phase, as a higher resting HR in the high hormone phase has been commonly observed in both naturally cycling females and those using hormonal contraceptives (Williams et al., 2024).

In a study by Ramesh and colleagues, the lack of a relationship between estradiol and HRV measured in the frequency domain in premenopausal females suggests that estradiol is likely not a key factor in modulating frequency domain metrics of HRV in females (Ramesh et al., 2022). Similarly, in exploratory analysis, we found no relationships between serum estradiol, progesterone, or levonorgestrel concentrations and any of our measures of HRV. Given that all females in this study were currently using an OCP2, endogenous hormones were low, and therefore it is not a strong model for determining the role of serum estradiol and progesterone concentration on HRV. However, from our results, we can speculate that levonorgestrel is not correlated with these HRV parameters. In contrast, a study conducted by Schmalenberger and colleagues found that higher‐than‐predicted levels of progesterone were correlated with lower‐than‐usual HRV in the mid‐luteal phase (Schmalenberger et al., 2020). This finding is in accordance with the hypothesis that progesterone may antagonize the beneficial effects of estradiol on cardiovascular and autonomic function (Stadler et al., 2019; Stone & MacDonald, 2023). Currently, it is unknown the mechanism by which endogenous estradiol and progesterone, or exogenous synthetic progestin, may influence HRV and other measures of autonomic function, presenting itself as an important avenue of future research.

### 4.1 Limitations

There are several limitations to be discussed. First, HRV is influenced by numerous physiological and behavioral factors. For example, HRV is sensitive to physiological changes in intrathoracic pressure, respiratory movements, chemoreflex, HR, stroke volume, BP, stress, anxiety, and physical activity, to name a few (van Ravenswaaij‐Arts et al., 1993). Although participants were instructed to maintain consistent breathing patterns during HRV measurements, respiratory rate was not directly measured or controlled, and therefore the influence of potential differences in respiration between visits cannot be excluded. Limitations to measuring HRV in the frequency domain have also been identified (Malik & Camm, 1993); for example, there is little sympathetic contribution to the LF band in a supine position, and overall many studies investigating HRV in humans do not use standardized approaches, making it difficult to compare results between studies (Laborde et al., 2017).

The unsupervised nature of the study (i.e., wearing the OURA® smart ring at home), although ecologically valid, likely resulted in increased variability in these nocturnal measurements. However, all participants were instructed to maintain the same lifestyle habits over the 2 weeks, and data from the OURA® smart ring revealed no change in sleep parameters or vigorous physical activity (Supplemental Table 1). Additionally, although participants were fitted with an OURA® smart ring sized for their non‐dominant index finger (or, when this was not possible, their non‐dominant ring finger) and were instructed to wear the ring on the same finger at all times, we did not verify compliance with these instructions throughout the week. Similarly, because the OURA® smart ring uses PPG, changes in peripheral blood flow could have influenced the rMSSD measurements. Furthermore, although the OURA® smart ring provides a readout of rMSSD, it measures pulse rate variability rather than HRV, which may be influenced by factors beyond cardiovagal activity, such as changes in peripheral vascular properties and total peripheral resistance. Lastly, as described previously, the OURA® interface only provides data in 5‐min segments, and beat‐by‐beat data is not available, limiting temporal resolution.

Our study is strengthened by including two methods of measuring HRV, stratifying by OCP generation and quantifying estradiol, progesterone, and levonorgestrel serum concentrations to confirm OCP phase, and counterbalancing visit order. Future research should investigate the influence of HRV between OCP phases in other popular generations, like OCP3 and OCP4, to determine if findings differ between OCP generation.

## 5 CONCLUSION

This study was the first to investigate the influence of OCP2 phase on metrics of HRV using both the OURA® smart ring and ECG monitoring. Our study found lower HRV in the time and frequency domain in the active phase of the OCP2 cycle, compared to the placebo phase. However, most of these differences were no longer apparent after covarying for resting heart rate. Future research must investigate whether differences in HRV observed across hormonal cycle phase are due to changes in cardiovagal tone or a result of a changing hemodynamic environment.

## AUTHOR CONTRIBUTIONS

J.C.S., J.C., and M.J.M. incepted this project. J.C.S, J.C., and M.I.B. contributed to data collection and analysis. J.C.S. created all figures and conducted statistical analysis with support from M.J.M. J.C.S. wrote the manuscript. All authors approved the final version of the manuscript.

## FUNDING INFORMATION

J.C.S. was funded by a Natural Sciences and Engineering Research Council (NSERC) Canadian Graduate Scholarship (Doctoral), and a Heart & Stroke Doctoral Personnel Awards for Women's Heart and/or Brain. Health. J.C.S., J.C., M.I.B., and M.J.M. were supported by a NSERC Discovery Grant to M.J.M.'s Vascular Dynamics Lab.

## CONFLICT OF INTEREST STATEMENT

None.

## ETHICS STATEMENT

This research study was approved by the Hamilton Integrated Research Ethics Board (project no. 16823).

## Supporting information


**Supplemental Table 1:** Additional OURA® smart ring parameters. Data are expressed as mean ± SD and averaged across the same days that root mean square of successive differences metrics were extracted. *n* = 16 for sleep variables, *n* = 14 for high activity met minutes.

## Data Availability

The datasets generated during and/or analyzed during the current study are available from the corresponding author on reasonable request.
