# Supplementary material for: Changes in central hemodynamics and heart rate variability across active and placebo phases in second‐generation oral contraceptive pill users
Source: Physiol Rep. 2026 Nov 9;14(21):e71175. doi: 10.14814/phy2.71175 (PMC13748814; doi:10.14814/phy2.71175)
Supplement: Supplementary file 1 [file PHY2-14-e71175-s001.docx]

**Supplemental Material**

*Supplemental Table 1:* Additional OURA^®^ smart ring parameters. Data are expressed as mean $\pm$ SD and averaged across the same days that root mean square of successive differences metrics were extracted. n = 16 for sleep variables, n = 14 for high activity met minutes.

|  | Placebo | Active | P | d |
| --- | --- | --- | --- | --- |
| Deep Sleep Duration (minutes) | 90 $\pm$ 14 | 87 $\pm$11 | 0.572 | 0.14 |
| Sleep Efficiency (%) | 87 $\pm$ 5 | 88 $\pm$ 4 | 0.495 | $-$0.17 |
| Sleep Latency (minutes) | 17 $\pm$ 10 | 16 $\pm$ 7 | 0.416 | 0.21 |
| Light Sleep Duration (minutes) | 236 $\pm$ 32 | 248 $\pm$ 37 | 0.295 | $-$0.27 |
| REM sleep duration (minutes) | 93 $\pm$ 19 | 92 $\pm$ 21 | 0.771 | 0.07 |
| Time in bed (minutes) | 475 $\pm$54 | 484 $\pm$ 58 | 0.625 | $-$0.12 |
| Total Sleep Duration (minutes) | 419 $\pm$ 46 | 426 $\pm$ 52 | 0.660 | $-$0.11 |
| High Activity Met Minutes | 56 $\pm$ 110 | 26 $\pm$ 32 | 0.214 | 0.36 |
